# ‘Bear Feels Sick’: A Systematic Review of Picture Books About Physical Symptoms in Dutch Children's Literature

**DOI:** 10.1111/cch.70069

**Published:** 2025-03-20

**Authors:** Sterre van der Ziel, Elske Hogendoorn, Brian A. Jorge, Marijn W. G. van Dijk, Michel J. van Vliet, Judith G. M. Rosmalen

**Affiliations:** ^1^ Department of Psychiatry University of Groningen, University Medical Center Groningen Groningen the Netherlands; ^2^ Nieuwenhuis Institute for Educational Sciences University of Groningen Groningen the Netherlands; ^3^ University of Groningen, University Medical Center Groningen, Beatrix Children's Hospital Groningen the Netherlands; ^4^ Department of Internal Medicine University of Groningen, University Medical Center Groningen Groningen the Netherlands

**Keywords:** health communication, physical symptoms, picture books, systematic review, young children

## Abstract

**Background:**

Young children frequently experience physical symptoms, such as common cold, vaccination pain or a playground injury. Picture books about physical symptoms and illness are a source of information for young children. This study assessed the nature and causes of and responses to physical symptoms depicted in Dutch children's picture books.

**Methods:**

A systematic search for Dutch picture books for children between the ages of 2 and 8 years about physical symptoms or illness was conducted in public library catalogues in the Netherlands, with terms such as ‘pain’ and ‘doctor’. Only picture books with a fictional storyline, in which the main character experienced symptoms or illness, were included. A content analysis was conducted by two independent reviewers.

**Results:**

Fifty‐five books met the inclusion criteria. The most common symptoms were injuries (29%), cold symptoms (21%), fatigue/malaise (14%) and fever (11%). Causes were mostly unknown (41%), due to accidents (38%) or infections (18%). Accidents were always caused by the main character. In 89% of the picture books, remedies were necessary to resolve the symptoms, including bandages (36%), bedrest (33%), medical interventions (33%) or medication (27%). Fifty‐six percent of the books depicted seeking medical care. Four themes regarding the morals of the stories were identified: the seemingly scary hospital or doctor, the responsibility of the main character to engage in coping strategies, the importance of social contacts, and illness gains after possible unpleasantness of symptoms.

**Conclusions:**

The picture books about physical symptoms and illness in our sample depicted various symptoms, limited causes and a range of responses. Physical symptoms and illness were rarely normalized in the stories, often requiring medical intervention rather than spontaneous resolution.

**Summary**

- Picture books about physical symptoms and illness are a source of information for young children and can be used to facilitate conversations about this topic.
- This systematic review found that Dutch picture books for children aged 2–8 years depict various symptoms, of which causes are mostly unknown or due to accidents that were the main character's own fault.
- Physical symptoms and illness are rarely normalized in the stories.
- Being scared of the hospital or doctor is often mentioned in the books, whereas it may be better to focus on the positive aspects to prevent unnecessary fear in children with little experience with the healthcare system.
- There is a lack of picture books that portray physical symptoms as a natural part of life, which in most cases resolve spontaneously without medical intervention.

## 1 Introduction

Young children encounter a variety of pains and other physical discomforts on a regular basis in their lives (Baeyer, Baskerville, and McGrath 1998; Fearon, McGrath, and Achat 1996). These experiences with physical symptoms may vary from small injuries in the playground to symptoms caused by viral infections. From an early age onwards, children learn how to interpret and respond to these everyday symptoms. These early‐life learning experiences possibly lay the foundations for how individuals cope with symptoms throughout life (Fotopoulou and Tsakiris 2017). Children learn from their caregivers how to approach experiences with physical symptoms or illness. According to the social learning theory, these social interactions with caregivers may shape the way children cope with physical symptoms in three ways (Levy, Langer, and Whitehead 2007; Bandura and Walters 1977). Firstly, through parents' responses to their child's symptoms, the child experiences direct consequences of expressing their symptoms. Secondly, children learn by observing parental responses to parents' own symptoms and the consequences of these responses. Thirdly, children talk with parents about symptoms and illness in everyday situations and thereby learn to give words to physical symptoms and illness experiences.

Research shows that children as young as 4 years old are able to talk about their symptom experiences (Esteve and Marquina‐Aponte 2012; Franck, Sheikh, and Oulton 2008; Franck, Noble, and Liossi 2010a; Van der Ziel et al. 2022). Besides their own experiences, children may also learn from discussing the experiences of others, such as fictional characters in picture books, TV shows or movies. For parents who want to discuss the topic of physical symptoms and illness with their children, picture books can be a helpful tool. Reading picture books together is a light‐hearted way of addressing topics that children are facing, and which, by creating a safe distance, can facilitate conversations about these topics (Corr 2006). Moreover, it is a way of communication that fits the cognitive level and perception of the world of young children (Lefevre 2010). Reading picture books together thus provides a learning opportunity for the child. Indeed, children's books have proven useful in providing information, education and support regarding cancer, surgery and Alzheimer's disease (Bugge, Helseth, and Darbyshire 2009; Felder‐Puig et al. 2003; Sakai, Carpenter, and Rieger 2012). Picture books are also widely used in preschool and primary school, primarily for educational purposes (Mol, Bus, and Jong 2009; Zhang, Sun, and Yeung 2023).

An earlier study examined the content of English picture books related to illness, injury and health (Turner 2006). This study included a larger sample of 119 books published between 1969 and 2003, providing an overview of symptoms, their possible causes and their consequences. Although the study listed descriptive examples of symptom types and their consequences, it did not indicate how frequently these appeared in the books. The study also identified caregivers depicted in the books (mothers, fathers, healthcare professionals or others) but offered no further details on caregiving behaviours. More insight into the frequency of symptom types, causes and responses in picture books would be beneficial. Such information could shed light on what children are exposed to through picture book literature and, thus, what they may learn from it. Additionally, a more detailed analysis of symptom responses, both by the child experiencing symptoms and by family members, friends and/or healthcare professionals, would be valuable for a deeper understanding. Furthermore, the previous English study included several books about serious diseases, such as cancer and the plague. It is still unclear if picture books also portray stories about common everyday physical symptoms. These picture books could be beneficial because they reflect what children experience frequently and therefore relate to children's day‐to‐day life experiences. However, prior research on pain depictions in movies and television shows for young children shows an underrepresentation of everyday pains compared to injury and violent pain (Mueri et al. 2021).

The aims of this systematic review were therefore (1) to provide an overview of picture books about physical symptoms and illness available in the Dutch children's literature and (2) to investigate the depiction of the nature and causes of these symptoms and illnesses, the responses to symptoms and illness and the moral of the story.

## 2 Methods

### 2.1 Search Method and Book Selection

A systematic search was performed in the catalogue of the 34 public libraries of the province of Groningen in the Netherlands, including libraries in both urban and rural areas. As this research was not a systematic review of scientific articles, the review was not formally registered. First, search terms were formulated in a multidisciplinary team consisting of a paediatrician, a professor of pedagogy, a professor of psychosomatic medicine and two PhD students, S.Z. and E.H., with respective backgrounds in medicine and pedagogy. The search terms (in Dutch) consisted of symptoms and terms concerning health, illnesses and medical care. When relevant, synonyms of terms were added based on the books that were identified in the search. In the search, terms were used with an asterisk to include supplementary terms and conjugations (e.g., ‘*ache’ targeting ache, headache and stomachache or ‘blood*’ targeting blood and bloody). A complete list of the search terms can be accessed via the website of the Open Science Framework (Van der Ziel and Hogendoorn 2024). The search terms were used in the library catalogue to find matches with title or subject, using a filter for age (0 to 8 years old) defined by the library. The initial search was conducted in 2022, with additional searches in February and November 2023 to add newly published books.

After the search was conducted, all duplicates were removed from the dataset. Subsequently, the titles and short descriptions of all books were screened for eligibility by 11 undergraduate medicine students, supervised by S.Z. and E.H.. To meet the inclusion criteria for full‐text analysis, the books had to be picture books intended to be read together with an adult, published in Dutch, covering the topic of physical symptoms or illness, and targeting an audience of 2‐to‐8‐year‐old children. Furthermore, only books were included in which the main character of the story experienced physical symptoms or an illness, and not, for example, only a parent or friend. Only books with a fictional storyline were included, which meant that informational books with a solely educational purpose were excluded, such as books that explain the objects in a hospital. Books for children to read on their own were excluded as well. As one of the aims was to assess the current availability of picture books about physical symptoms and illness, there was no exclusion based on year of publication.

### 2.2 Analysis

A content analysis was conducted on all included books. This analysis was of a quantitative nature, complemented by a qualitative item. A coding scheme was developed based on previous research that focused on young children's experiences with everyday physical symptoms, previous reviews of children's literature and group discussion with professionals in the field of physical symptoms and child development (Van der Ziel et al. 2022; Arruda‐Colli, Weaver, and Wiener 2017; Huang et al. 2015). This coding scheme consisted of five general items about the title, author name, author background, year of publication and publisher. Furthermore, 25 quantitative items concerning the nature, cause of and responses to the symptoms were included in the coding scheme, as well as one qualitative item about the moral of the story. As a pilot, two PhD students (S.Z. and E.H.) analysed three books using the coding scheme and made some final adjustments. After the pilot, all picture books were independently double coded by two undergraduate students. After coding each book, the results were compared and disagreements were discussed face‐to‐face. In case the two reviewers did not reach consensus, a third reviewer (S.Z. or E.H.) was consulted to resolve the disagreement.

Interrater reliability was calculated between the two coders using percentage of agreement for the 30 quantitative items of the checklist, excluding the general items and the qualitative question about the moral of the story. If the percentage of agreement was below 75%, S.Z. or E.H. coded the book as a third coder, which happened for 10% of the books. For the qualitative item, discussion between coders was used to reach consensus and the results of the discussion were reviewed by S.Z. and E.H. Approximately 50% of the books were read by both S.Z. and E.H. to check whether they extracted a similar moral of the story.

After coding all picture books, the results of the quantitative items were grouped into larger categories by S.Z. and E.H. for analysis purposes. The qualitative outcomes underwent analysis using the One Sheet of Paper (OSOP) method (Ziebland and McPherson 2006). The OSOP method is an analysis method in which extracted data are visually rearranged to define themes. All individual outcomes on the qualitative item about the moral of the story were inspected and written on a large piece of paper, while arranging the morals based on differences and similarities. After adding all morals to the OSOP, the visual arrangement was reviewed to identify associations. The outcomes of the OSOP method and possible themes were discussed in the team.

**FIGURE 1:**
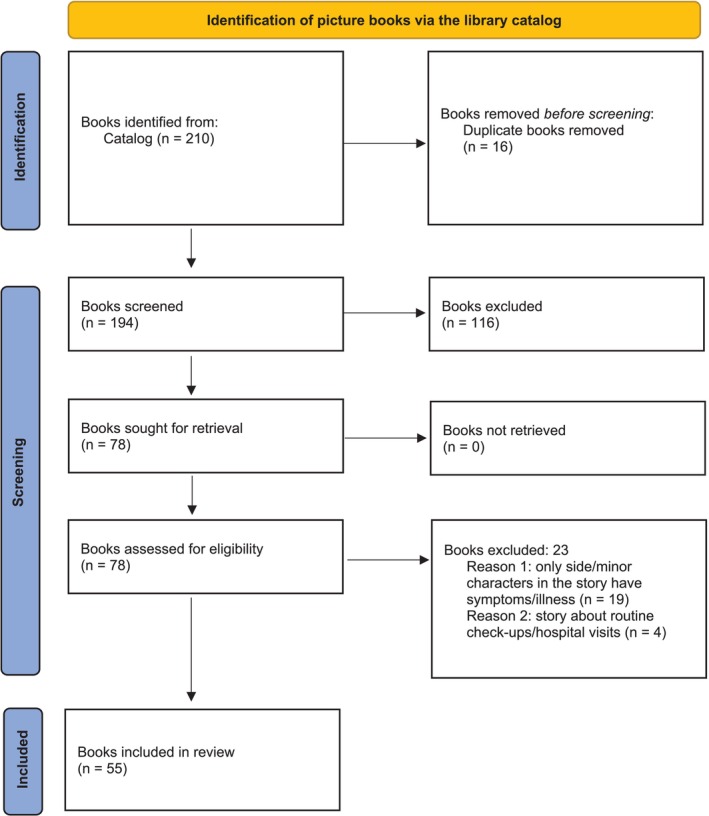
PRISMA 2020 flow diagram.

The coding scheme, search terms, list of included books and other supplementary material can be accessed via the website of the Open Science Framework (osf.io/f7mbd/files) (Van der Ziel and Hogendoorn 2024). The used data are available upon reasonable request. The PRISMA 2020 statement for reporting systematic reviews was followed in Figure 1 (Page et al. 2021).

## 3 Results

### 3.1 Descriptives

A total of 55 books were included in the content analysis, as is shown in the PRISMA flow diagram (Figure 1). Overall interrater reliability was excellent (*M* = 92%; median = 94%; range: 77%–100%). The year of publishing the first edition in Dutch ranged from 1976 to 2023 (median 2015). Most of the books were published between 2011 and 2015. The majority of the books were originally published in Dutch (*n* = 37 [67%]), followed by English (*n* = 13 [24%]) and German/French (*n* = 5 [9%]).

The main characters were animals in 33 of the included books (60%) and humans in 21 books (38%); in one book (2%), the main character was a vehicle. Most of the main characters were male (64%), a smaller proportion were female (35%) and in one book, there were multiple main characters of different sexes. In 40% of the books, the main character was the only character with physical symptoms or illness, whereas in 60% of the books, friends, relatives, healthcare professionals or stuffed animals also experienced symptoms.

### 3.2 Symptoms

**FIGURE 2:**
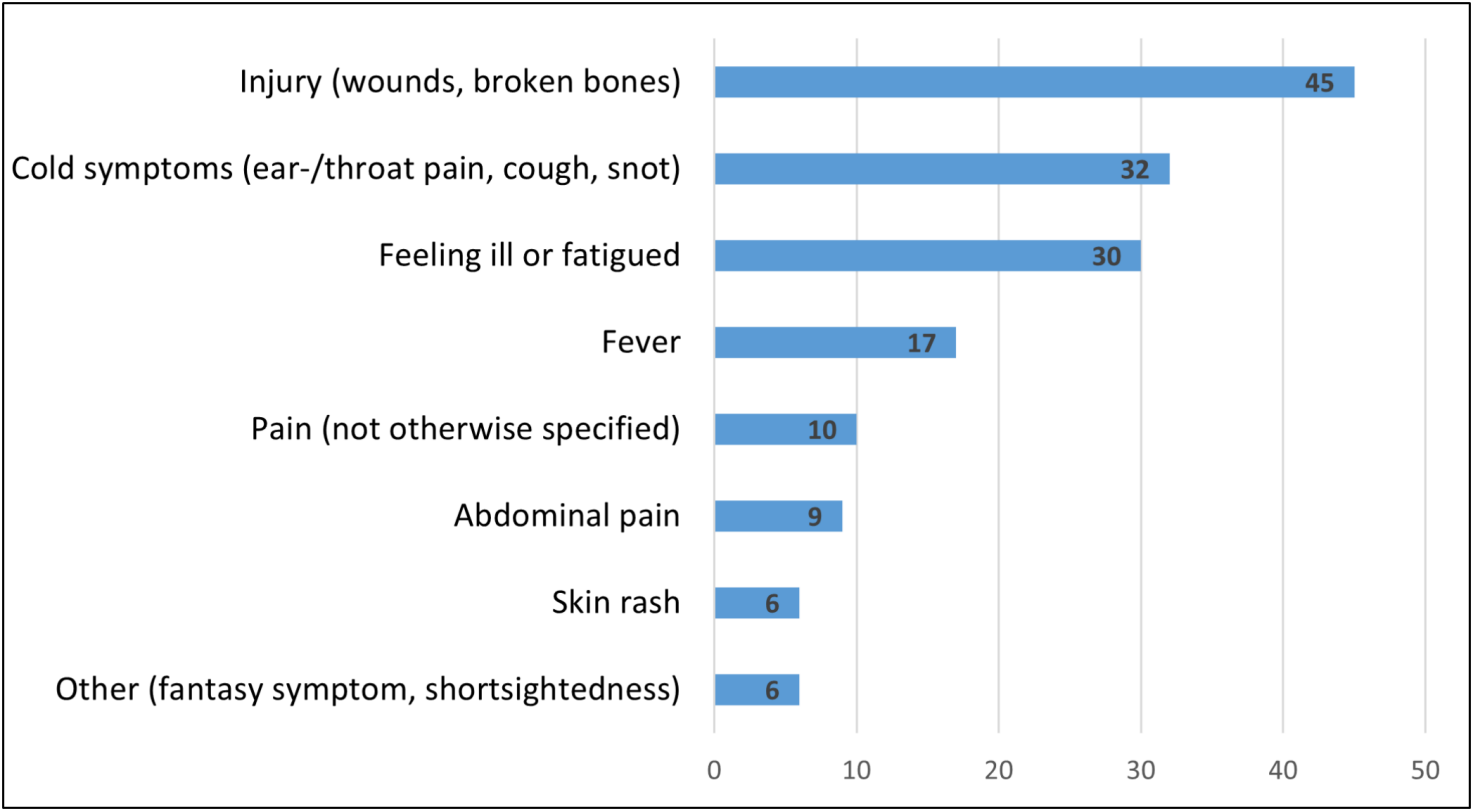
Described physical symptoms*. * Not all physical symptoms were experienced by the main character of the story.

Across the books, a total of 155 physical symptoms were described, with most books depicting multiple symptoms in one story. Many different descriptions of physical symptoms were used. For example, fever was described as ‘being hot and cold’, ‘feeling hot’ or ‘shivering’. For the analysis, the symptoms were grouped into larger umbrella terms. The most frequently described symptoms were injuries (29%), cold symptoms (21%) and feeling ill (14%). Figure 2 provides an overview of the described physical symptoms.

### 3.3 Causes

Nearly half of the picture books did not specify the causes of the physical symptoms (*n* = 21 [41%]). Most mentioned causes were accidents (38%) and infections (18%). Rare causes were chemotherapy (2%), and the main character pretending to have the symptoms (2%).

The books in which accidents happened usually made clear whose fault the accident was: The main character was always the one to blame for the symptoms, with causes such as falling from ‘running on the stairs’ or ‘jumping too high on the trampoline.’ Only three books about infections gave insight into whose fault the symptoms were, with two books about food poisoning (i.e., the main character sneakily eating suspicious food) and one book about a viral infection (i.e., not wearing a coat in the rain and thus getting a cold).

### 3.4 Responses

Characters in the picture books showed different, often multiple responses to the physical symptoms (Table 1).

**TABLE 1:** Main character responses to physical symptoms.

Type of behaviour/reactions	Number (%^a^) of books in which the response was mentioned
Withdrawal behaviour	25 (45%)
Verbally expressing their symptoms	18 (33%)
Seeking physical comfort	13 (24%)
Crying	11 (20%)
Continuing normal activities (or trying to)	6 (11%)
Seeking (medical) help	5 (9%)
Being angry	3 (5%)
Unknown/no specific response	3 (5%)

^a^ As the characters in the stories often showed multiple behaviours in response to symptoms, the percentages add up to more than a 100%.

The responses to the main characters' symptoms from relatives or friends were very positive, and many different behaviours were mentioned in the books. Friends or family took care of the main character (78%), paid special attention or visits (64%), sought medical care (49%), gave presents (40%) or granted special privileges (15%). In only two books there was no clear response from relatives or friends.

The symptoms of the main character seldom resolved spontaneously: In 89% (*n* = 49) of the books, a remedy was necessary. In total, 97 remedies were mentioned in the books, with most books identifying multiple remedies. The mentioned remedies are shown in Table 2.

**TABLE 2:** Mentioned remedies for physical symptoms.

Type of remedy	Number (%) of books in which the remedy was mentioned (%)
Bandage, plasters or casts	20 (36%)
Rest or sleeping	18 (33%)
Medical intervention (e.g., operations)	18 (33%)
Medication or ointments	15 (27%)
Food or drinks	13(24%)
Love and care	6 (11%)
None	5 (9%)
Other (horseback riding, magic spells)	2 (4%)

In 56% (*n* = 31) of the picture books, medical care was sought for the symptoms or illness of the main character, either initiated by the main character, other characters or both. Often, more than one healthcare professional was depicted in the book. The majority of the 35 doctors in the stories were male (*n* = 24 [69%]). All 15 nurses in the stories were female. The male healthcare professionals were described as skilled or competent in 46% of the cases, compared with 20% of the female healthcare providers. Female healthcare professionals were more often described as nice and sweet (44%) or caring (16%), compared with the male healthcare professionals in the stories, who were described as nice in 35% of the cases, but never as caring (0%).

The most frequently described action taken by healthcare professionals in the picture books was providing medical interventions, such as operations (51%), followed by giving explanations about the symptoms or illness (33%), performing a physical exam or other diagnostic tests (25%) and/or giving reassurance (22%). Many books included multiple actions from healthcare professionals.

### 3.5 Morals

Although the picture books contained various messages, four themes regarding the moral of the story emerged from the data. The first important theme, corresponding to numerous, often similar, morals, included the message that the doctor or hospital may seem scary, but it is important to go and you will be treated well. The second theme focused mainly on coping strategies that were presented as a responsibility of the person feeling sick or having symptoms: things one ‘has’ to do when sick or in pain. Often described coping strategies were listening/obeying to the doctor and taking rest or medication. The third theme that emerged from the data is the importance of social contacts: Friends will take care of you and, conversely, you have to take care of your sick friends. The last theme that was found, although less prominent, was that of illness gain. Often, the moral expressed something that was unpleasant (e.g., being in hospital and having to stay in bed), but that there was some benefit alongside (receiving attention, sweets or presents).

Two morals seem to be outliers compared to the main themes. One moral described the healing powers of animals compared with the regular healthcare system, whereas another moral from a picture book about chronic illness emphasized the importance of focusing on the possibilities within your illness.

## 4 Discussion

Our findings show a varied depiction of symptoms in Dutch picture books, with the majority of books depicting physical injuries. The causes of symptoms were mostly unknown or linked to accidents or infections. When describing injuries due to accidents, the main character was always the one to blame. With regard to symptom responses, the main character mostly showed withdrawal behaviour or verbal expression of symptoms or sought physical comfort. Relatives or friends of the main character often showed behaviours such as caring, paying special attention or seeking medical care. Nearly all physical symptoms required a remedy; only a few resolved spontaneously. Often, medical care and healthcare professionals were described, with mostly male doctors and only female nurses. The morals of the stories from the picture books showed four themes: the seemingly scary hospital or doctor (where the main character is treated well), the responsibility of the main character to engage in coping strategies, the importance of social contacts, and the illness gains after possible unpleasantness of symptoms.

The one earlier study on physical symptoms and illness in picture books that we identified was conducted in the English children's literature (Turner 2006). This sample included mainly older books (most books published between 1990 and 1999) than the books in our sample (most books published between 2011 and 2015). Although the focus was somewhat different compared with the current study, this previous study provides an overview of the characters, symptoms and possible causes. In the English sample, injuries were only described in 8% of the picture books, whereas these were the most frequently described symptoms in our sample, occurring in 29% of the books. Moreover, the causes of the symptoms were even less frequently mentioned in the English sample, only in 35% of the books, compared with 59% in our sample. Although in 24% of the books in our sample, healthcare professionals did give explanations about the symptoms or illness, this concerned mainly explanations about the remedy (e.g., explaining that an operation or rest is necessary), rather than the cause. This is remarkable because multiple studies have shown that children as young as 4 years old already think and talk about the causes of symptoms (Van der Ziel et al. 2022; Kalish 1996). One of those studies reported that young children mostly suggest everyday reasons related to normal body functions for symptoms, such as hunger or eating too much (Van der Ziel et al. 2022). Not including causes in the stories diminishes the potential to educate children and engage them in conversations about this topic. Since causes often offer leads for resolving the symptoms, mentioning causes in books could invite children to think about appropriate solutions when they experience symptoms themselves. Moreover, including normalizing causes of symptoms, thus framing symptoms as common physiological experiences, may align more closely with children's experiences and reduce fear around health issues. Interestingly, nerves, stress or excitement are not once mentioned in our sample as causes for symptoms, whereas these are common causes in children (Hietanen et al. 2016).

When causes were mentioned in the picture books of this study, they mostly involved accidents in which the main character was the culprit. This may be a reflection of the Western‐European backgrounds of the authors and suggest an intentional pedagogical strategy aimed at teaching children moral character, illustrated by the ‘Chickens come home to roost’ message (meaning that irresponsible actions will ultimately lead to trouble for the person who initiated them). An observational study on minor injuries in everyday life found that young children do indeed cause the majority of everyday pain incidents themselves (Noel et al. 2018). However, the majority of injuries in the picture books in our sample necessitated a (partly) unpleasant medical procedure. This might teach children that they need to play carefully because otherwise they could hurt themselves, possibly even leading to a hospital visit. Vigorous physical play, however, involving running, climbing and jumping, is important for motor skill acquirement and allows children to test the boundaries of their strength and assess dangers (Little and Wyver 2008). Indeed, risky play has been associated with numerous positive health outcomes, and in case risky play leads to incidents, these usually result in minor injuries requiring minimal or no medical treatment (Brussoni et al. 2015). Therefore, picture books highlighting the advantages of physical play and showing only minor play‐related injuries that do not require medical intervention may better reflect reality and encourage children to engage in beneficial play behaviours.

The responses to physical symptoms that were depicted in the books from our sample correspond well to the existing literature on actual children's behaviour when experiencing symptoms or feeling sick. The most frequently occurring behaviours in the picture books were withdrawing behaviours, seeking (physical) comfort from a loved one and verbally expressing symptoms. These behaviours have been described by parents, as well as paediatric professionals and preschoolers themselves (Franck, Sheikh, and Oulton 2008; Van der Ziel et al. 2022; Franck, Noble, and Liossi 2010b; Van der Ziel et al. 2024).

With regard to the morals of the stories, an important theme concerned the seemingly scary hospital. Indeed, fear of the hospital is frequently reported by children (Salmela, Salanterä, and Aronen 2009). It is nonetheless remarkable that words like ‘scary’ are used in descriptions of the hospital or medical procedures in the books, since the literature implies the use of such language in medical contexts, even when used in a reassuring manner (e.g., ‘The hospital is not scary’ and ‘Do not worry’), may actually induce anxiety (McMurtry et al. 2010; Fusco et al. 2020). It has been suggested that such reassuring utterances serve as a signal of fear to children, communicating to them that there is in fact something to be scared of (Chambers, Craig, and Bennett 2002). It is recommended that parents and healthcare professionals rather use distraction and suggestive language focused on a positive outcome (Siva 2022; Geagea et al. 2023; Lang et al. 2005). Picture books may aid in this by depicting only positive aspects of the hospital and healthcare professionals, without mentioning that the hospital is scary. After all, healthy children may not yet have any experience with hospital visits, and therefore, no expectations about the hospital being scary or bad.

Research on the experience of physical symptoms in young children shows that children report social contacts and caregiving by loved ones as important when having physical symptoms, as well as describing possible benefits of having symptoms, which is in line with the themes socials contacts and illness gains that emerged in our study (Franck, Sheikh, and Oulton 2008; Van der Ziel et al. 2022).

### 4.1 Strengths and Limitations

This study has a number of strengths. The search terms were formulated in a multidisciplinary team, which enhanced the comprehensiveness of the terms. We conducted the search in public libraries, thereby including picture books that are widely available and generally free to borrow for parents and children in the region. The public libraries in the whole province of Groningen were covered, thus including 34 libraries in urban as well as rural areas. Furthermore, overall interrater reliability was excellent, and disagreements were dissolved by discussion with an independent third reviewer. Another strength is the integration of both quantitative and qualitative items in the content analysis. This allowed us to provide a clear overview of both occurrence and distributions of aspects of interest such as symptom nature and duration, as well as to describe more in‐depth the sentiment or ‘lessons’ to take away from the books.

Some limitations should be considered as well. The search was performed in the public library catalogue of the province of Groningen, and library book selection may slightly vary by region. Hence, there may be other eligible picture books that were not included in this systematic review. In addition, we were limited to books that were originally written in or translated into Dutch. The stories of the books in our sample mainly reflect the Western‐European culture and were all written by Western authors. Books from non‐Western cultures could depict different descriptions of symptoms, causes, responses, remedies and medical care than the books in the current sample. Another limitation concerns the extraction of the stories' morals by the researchers themselves. This increases the risk of subjectivity, since the researchers' own backgrounds and beliefs may have shaped their perceptions of the stories. However, every book was double coded, followed by face‐to‐face discussion, enriching the variety of perspectives and interpretations of these morals.

### 4.2 Implications and Future Directions

The current systematic review focused on picture books in which the main character of the story experienced physical symptoms or an illness. Stories in which family members experience symptoms might depict symptoms of a different nature than the symptoms that we found. Future studies may include such books to provide a clear overview of this. By focusing on symptoms and symptom responses of parents, such studies may give more insight into social learning of symptom‐related behaviour through the use of media. Educational books may also be included in future research, to obtain a complete overview of what young children may learn from the content of picture books about physical symptoms. Such books may be preferred less for reading together but may be used more in schools or daycare. Video‐observation studies on parents and children reading picture books about symptoms together could clarify in what ways parents convey the stories to children and what kind of parent–child interactions about this topic occur while reading. Furthermore, the depiction of physical symptom types, causes and responses in screen media warrants investigation, as this source plays an increasingly prominent role in children's social learning (Richert, Robb, and Smith 2011).

Many of the books from our sample give little explanation about causes of symptoms, with none of the books mentioning stress or excitement as a possible cause. Moreover, most available picture books concern physical symptoms that do not spontaneously disappear and require a remedy, often involving a medical intervention. This indicates that there is a lack of picture books that portray physical symptoms as a natural part of life, which in most cases are transient and resolve without medical intervention (Malas et al. 2017). Such books may teach children that most of the time, they can largely continue their daily activities when experiencing symptoms. As fictional picture books often seem to have an underlying educational purpose, future picture books about physical symptoms may benefit from incorporating these normalizing messages in their story. Doing so would improve consistency between education and current recommendations from scientific literature.

## Author Contributions

**Sterre van der Ziel:** conceptualization, writing – original draft, investigation, methodology, formal analysis, supervision, data curation, resources, project administration. **Elske Hogendoorn:** conceptualization, investigation, writing – original draft, methodology, data curation, supervision, resources, formal analysis, project administration. **Brian A. Jorge:** writing – review and editing, investigation, resources. **Marijn W.G. van Dijk:** writing – review and editing, conceptualization. **Michel J. van Vliet:** writing – review and editing, conceptualization. **Judith G.M. Rosmalen:** conceptualization, funding acquisition, writing – review and editing, methodology, supervision.

## Conflicts of Interest

The authors declare no conflicts of interest.

## Data Availability

The data that support the findings of this study are available from the corresponding author upon reasonable request.
